# Semaphorin-3 Promotes Specific Immunotherapy Effects on Experimental Food Allergy

**DOI:** 10.1155/2022/5414993

**Published:** 2022-06-19

**Authors:** Huang Huang, Yu Liu, Yanan Wang, Huazhen Liu, Bailing Xie, Michael B. W. Zheng, Liteng Yang, Qinmiao Huang, Yongjin Wu, Gaohui Wu, Pengyuan Zheng, Pingchang Yang

**Affiliations:** ^1^Department of Gastroenterology, Fifth Affiliated Hospital of Zhengzhou University, Zhengzhou, China; ^2^Department of General Practice, The Third Affiliated Hospital of Shenzhen University, Shenzhen, China; ^3^Guangdong Provincial Key Laboratory of Regional Immunity and Diseases, Shenzhen, China; ^4^Institute of Allergy & Immunology, Shenzhen University School of Medicine, State Key Laboratory of Respiratory Disease Allergy Division at Shenzhen University, Shenzhen, China; ^5^Department of Life Science, McMaster University, Hamilton, ON, Canada; ^6^Department of Respirology, The Third Affiliated Hospital of Shenzhen University, Shenzhen, China; ^7^Longgang ENT Hospital & Shenzhen ENT Institute, Shenzhen, China

## Abstract

Sustaining higher frequency of mast cells in the allergic lesion site has been recognized. Factors causing high numbers of mast cells in the local tissues are not fully understood yet. RAS signaling plays a role in sustaining certain cell activities. This study is aimed at elucidating the role of RAS activation in the apoptosis resistance induction in mast cells and at employing semaphorin 3A to regulate RAS activities in sensitized mast cells and alleviating the allergic response in the intestine. A food allergy (FA) mouse model was developed. Mast cells were isolated from FA mouse intestinal tissues by flow cytometry. Mast cell apoptosis was assessed by staining with annexin V and propidium iodide. We found that aberrantly higher p21-activated kinase-1 (Pak1) expression in FA mast cells was associated with mast cell aggregation in the intestine. Sensitization increased Pak1 expression and apoptosis resistance in intestinal mast cells. RAS and Pak1 mutually potentiated each other in sensitized mast cells. Semaphorin 3A (sema3A) suppressed the Pak1 expression and RAS activation in mast cells. sema3A restored the apoptosis sensitivity in sensitized mast cells. Administration of sema3A potentiated allergen-specific immunotherapy in experimental FA. In conclusion, mast cells of FA mice showed higher Pak1 expression and high RAS activation status that contributed to apoptosis resistance in mast cells. Administration of sema3A restored the sensitivity to apoptosis inducers and promoted the therapeutic effects of specific immunotherapy on experimental FA.

## 1. Introduction

Mast cells are one of the major effector cells in allergic response [1]. Specific IgE binds to the high affinity IgE receptors on the surface of mast cells to sensitize mast cells. Reexposure to specific antigens induces sensitized mast cells to degranulate; allergy mediators, such as histamine, serotonin, and leukotrienes, are released from mast cells to the local tissues and, thus, initiate the allergy attacks. It is recognized that a large number of mast cells gather in the local tissues with allergic lesions [1], while the mechanism by which mast cells gather at lesion tissues is not fully understood yet. On the other hand, the allergy-induction capacity is impaired in mice with mast cell deficiency [2]. This emphasizes the importance of mast cells in the pathogenesis of allergic diseases since mast cell-derived IL-4 plays a critical role in the initiation of aberrant Th2 responses [3].

Our previous studies showed apoptosis resistance in intestinal mast cells of mice with experimental food allergy (FA) [4]. Those mast cells expressed higher levels of Bcl2L12, one of the apoptosis inhibitors. The expression of Bcl2L12 conferred on mast cells the apoptosis resistance property. Blocking the Bcl2L12 expression could restore the ability to respond to apoptosis inducers in mast cells of FA mice [4]. However, factors and mechanism inducing sustained Bcl2L12 expression in mast cells are not clear.

The p21-activated kinases (Paks) are a family of serine/threonine kinases activated by Cdc42 (cell division control protein 42) and Rac1 (Ras-related C3 botulinum toxin substrate). They were discovered as binding proteins of small GTPase in rodent brain tissue [5]. Pak1 is localized to the cellular and nuclear membrane. Pak1 prevents cell apoptosis through the regulation of several signaling pathways, including the Raf1 pathway, Akt pathway, and Forkhead box pathway. Raf1 protects cells from apoptosis by stimulating complexes between Raf1 and B-cell lymphoma 2, as well as inhibiting the Bcl2-associated death promoter [6].

The semaphorin family has soluble and membrane-bound proteins that are associated with angiogenesis, neuronal development, cancer progression, and organogenesis [7]. Semaphorins also play a role in immune responses [8]. Various semaphorin members promote the immune response [9], whereas for others, the secreted semaphorins of class 3A (sema3A) negatively regulate immune responses [10]. sema3A can be produced by epithelial cells [11]. It is reported that administration of sema3A alleviates experimental allergic conjunctivitis [12]. Whether sema3A regulates mast cell lifespan, such as the sensitivity to apoptosis inducers, remains to be investigated. Published data indicate that aberrant RAS activation plays a critical role in the sustaining growth of cancer cells [13]. It is also recognized that RAS activation involves mast cell activities, such as in the allergic mediator release [14]. Since mast cells are the important effector cells in allergy attacks, modulating mast cell activities may also attenuate or inhibit allergic response. In this study, the role of RAS activation in the development of apoptosis resistance in mast cells was investigated in both cell experimental studies and FA animal model studies. The results showed that RAS activation indeed contributed to the development and maintenance of apoptosis resistance in mast cells of mice with FA. Administration of sema3A efficiently promoted specific immunotherapy in alleviating experimental FA.

## 2. Materials and Methods

### 2.1. Ethical Statement

The animal experimental procedures were approved by the Animal Ethical Committee at Zhengzhou University.

### 2.2. Isolation of Mast Cells from Mouse Intestine

Small intestinal segments were excised and cut into small pieces about 2 × 2 × 2 mm in size. The tissues were incubated with collagenase IV (0.5 mg/ml) for 30 min at 37°C with mild agitation. Single cells were filtered through a cell strainer (70 *μ*m first, then 40 *μ*m) and collected by centrifugation (800 × *g*, 5 min). With CD117 and FceRI as cell markers, mast cells were purified via flow cytometry (FCM). The purity of isolated mast cells was 95%-97% as checked by FCM. The viability of isolated mast cells was 97%-99% as assessed by the Trypan blue exclusion assay.

### 2.3. Assessment of RAS Activation in Mast Cells

The RAS activation status in mast cells was assessed by the RAS-specific ELISA with specific RAS activation assessing kits following the manufacturer's instructions.

### 2.4. Allergen-Specific Immunotherapy (AIT)

Following published procedures [15] with modification, FA mice were treated with a 2-week oral AIT starting one week after the last gavage with OVA. Briefly, mice were fed with OVA at the doses of 1 mg (days 1 and 2), 5 mg (days 3 and 4), 10 mg (days 5–7), 25 mg (days 8 and 9), and 50 mg (days 10–14) with or without mixing with sema3A (0.2 mg/mouse daily).

### 2.5. Collection of Gut Lavage Fluids (GLF)

Upon the sacrifice, a jejunal segment (15 cm) was excised and irrigated with 2 ml saline; the irrigation fluids were collected and centrifuged at 5000 × *g*; supernatant was collected and used as the GLF. Cytokine levels in GLF were determined by ELISA.

### 2.6. Statistics

The data are presented as mean ± SEM or median (IQR). The difference between two groups was determined by Student's *t* test or the Mann–Whitney test; ANOVA followed by Dunnett's test was performed among more than two groups. The Pearson correlation coefficient test was performed to determine the correlation between the two groups. *p* < 0.05 was set as a significant criterion.

### 2.7. Experimental Procedures Presented in the Online Supplemental Materials

Reagent sources, experimental procedures of mice, generation of bone marrow-derived mast cells, flow cytometry, real-time quantitative RT-PCR, Western blotting, enzyme-linked immunosorbent assay, immunoprecipitation, knockdown of plexin A2 or Sos1 expression in BMMCs by RNA interference, overexpression of Pak1 in BMMCs.

## 3. Results

### 3.1. Aberrantly Higher Pak1 Expression in FA Mast Cells Is Associated with Mast Cell Aggregation in the Intestine

**Figure 1:**
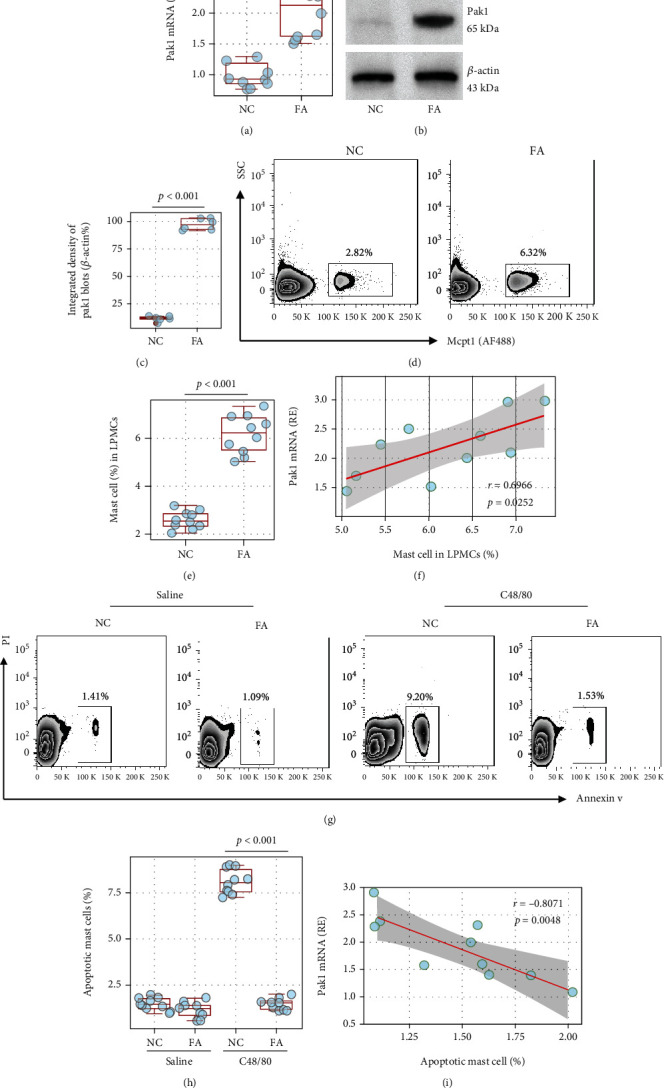
Pak1 is correlated with mast cell apoptosis resistance. (a–c) Mast cells were isolated from the mouse intestine and analyzed using RT-qPCR and Western blotting: (a) Pak1 mRNA levels; (b) Pak1 protein levels; (c) integrated density of Pak1 blots in (b). (d) Mast cell frequency in LPMCs (lamina propria mononuclear cells). (e) Positive correlation between Pak1 mRNA levels in mast cells and mast cell frequency in LPMCs. (g, h) Mast cells were isolated from LPMCs and exposed to C48/80 in the culture overnight. Gated FCM plots show apoptotic mast cell counts. Boxplots show summarized apoptotic mast cell counts. (i) Negative correlation between Pak1 mRNA levels in mast cells and the apoptotic mast cell counts. Each bubble in boxplots presents data obtained from one sample. The data of (b) are from one experiment that represents 3 independent experiments with pooled protein extracts from mast cells obtained from 10 experiments. NC: naïve control mice.

Our previous reports showed apoptosis resistance in FA mast cells (mast cells of FA mice) [4]; here, we took further insight into the underlying mechanism. Published data indicate that p21-activated kinase-1 (Pak1), a serine/threonine kinase, involves mast cell degranulation [16]. In an FA mouse model (Figure S1 in supplemental materials), we found higher Pak1 expression in FA mast cells (Figure S2 and Figures 1(a)–1(c)). The mRNA levels of Pak1 in FA mast cells were positively correlated with mast cell counts in the intestine (Figures 1(d) and 1(e)). The Pak1 mRNA levels in mast cells were positively correlated with mast cell frequency in the intestine (Figure 1(f)). We then isolated mast cells from the mouse intestine. The mast cells were exposed to C48/80 (a nonspecific mast cell activator) in the culture overnight. As analyzed by FCM, exposure to C48/80 induced much fewer apoptotic mast cells in the FA group than in the NC group (Figures 1(g) and 1(h)). A negative correlation was detected in the data between apoptotic cells and Pak1 mRNA levels in mast cells of the FA group (Figure 1(i)). Our previous studies show that Bcl2L12 plays a role in the apoptosis resistance of mast cells [4]. Thus, we also checked the expression of Bcl2L12 in mast cells. We found that the Bcl2L12 levels were also higher in mast cells isolated from the FA group than from the NC group. However, no correlation was detected between the Bcl2L12 levels and the mast cell frequency in LPMCs (Figure S2D-F). The results suggest that Pak1 may be associated with the apoptosis resistance in FA mast cells.

### 3.2. IgE Sensitization Induces Pak1 Expression and Apoptosis Resistance in Mast Cells

**Figure 2:**
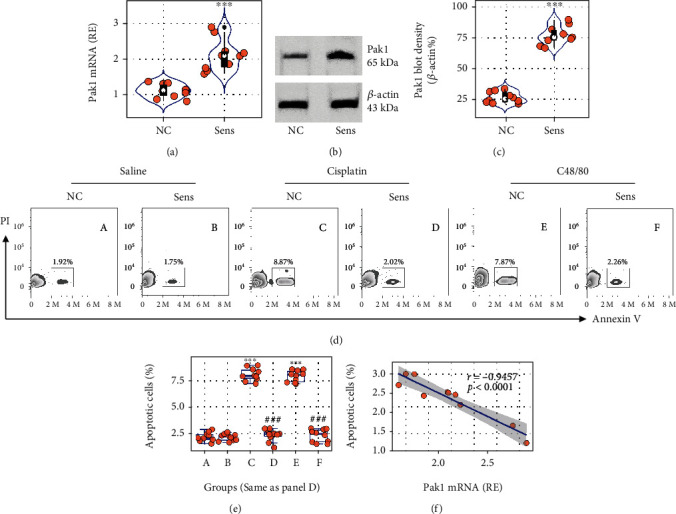
Assessment of the role of sensitization in Pak1 expression and apoptosis development in mast cells. (a, b) BMMCs were sensitized (Sens, in short) by exposing to OVA-specific IgE in the culture overnight. Violin plots show Pak1 mRNA levels, and immunoblots show Pak1 protein levels in BMMCs. (c) Integrated density of Pak1 blot density. (d, e) Sensitized BMMCs were stimulated with cisplatin (20 *μ*M) or C48/80 (0.1 mg/ml) in the culture overnight. Gated FCM plots show apoptotic BMMC cells. Boxplots show apoptotic BMMC frequency. (f) A negative correlation between Pak1 mRNA levels in sensitized BMMCs and the cisplatin-induced apoptotic BMMC frequency. Each bubble in the violin plots and boxplots presents data obtained from one experiment. ^∗∗∗^*p* < 0.001 (ANOVA+Dunnett's test), compared with the NC (naïve control) group. NC: Naïve control BMMCs.

The data of Figure 1 show higher a Pak1 level in FA mast cells, suggesting that sensitization may be an important factor in this event. To test this, BMMCs were prepared (Figure S3A) and exposed to OVA-specific IgE (Figure S3C) in the culture. The cells were then analyzed by RT-qPCR and Western blotting. The results showed that sensitization markedly increased the Pak1 expression in BMMCs (Figures 2(a)–2(c)). The cells were then exposed to cisplatin (a nonspecific apoptosis inducer) or C48/80 (a nonspecific mast cell activator that can induce the “activation-induced mast cell apoptosis” [4]) in the culture overnight and analyzed via FCM. We found that exposure to either cisplatin or C48/80 induced naïve BMMC apoptosis, but not in sensitized BMMCs (Figures 2(d) and 2(e)). A negative correlation was detected between the data of Pak1 mRNA levels and apoptotic cell frequency in sensitized BMMCs (Figure 2(f)). The data indicate that sensitization increases the Pak1 expression in BMMCs that may be associated with the development of apoptosis resistance in mast cells.

### 3.3. Pak1 Interacts with RAS in Sensitized Mast Cells

**Figure 3:**
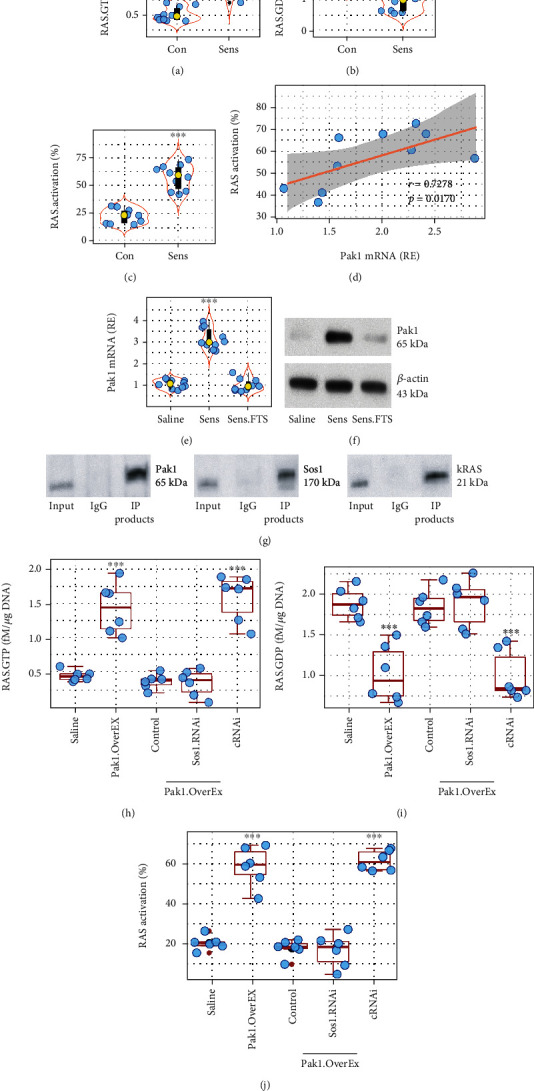
Assessment of the role of Pak1 in RAS activation in sensitized mast cells. (a–c) Sensitized (Sens) and naïve control (Con) BMMCs were analyzed by RAS-specific ELISA. Violin plots show RAS.GTP levels (a), RAS.GDP levels (b), and RAS activation status (c). (d) Positive correlation between RAS activation and Pak1 mRNA levels in sensitized BMMCs. (e, f) Pak1 mRNA (e) and protein (f) levels in sensitized BMMCs. FTS: a pan inhibitor of RAS in the culture (75 *μ*M). (g) A triple complex of Pak1, Sos1, and kRAS in sensitized BMMCs. (h–j) RAS activation in BMMCs after the treatment denoted on the *x* axis. Pak1.OverEx: BMMCs with Pak1 overexpression. Control: BMMCs were transfected with empty plasmids. Sos1.RNAi (cRNAi): BMMCs were treated with Sos1 RNAi (or control RNAi). The data in the violin plots and boxplots are presented as median (IQR). Each bubble presents data obtained from one experiment. ^∗∗∗^*p* < 0.001 [Student *t* test (a–c) or ANOVA+Dunnett's test (e, h–j)], compared with the saline group. Pearson correlation coefficient test was performed in (d).

As Pak1 can be activated by Ras-related small G-proteins [17], the high Pak1 expression implicates skewed RAS activation in sensitized BMMCs. To test this, RAS activation status in BMMCs was assessed. The results showed that sensitization markedly increased the RAS activation levels (Figures 3(a) and 3(c)) that was positively correlated with the Pak1 expression (Figure 3(d)). The sensitization-increased expression of Pak1 in BMMCs was blocked by the presence of FTS, a pan inhibitor of RAS (Figures 3(e) and 3(f)). The data implicate that Pak1 may interact with RAS.GTP-binding proteins to promote or potentiate RAS activation. To test this, proteins were extracted from sensitized BMMCs and analyzed via immunoprecipitation (IP) with anti-Pak1 Ab as bait. A triple complex of Pak1, Sos1, and KRAS was detected in the IP products (Figure 3(g)). Overexpression of Pak1 (Figure S4A) induced RAS activation in BMMCs that was abolished by knocking down the Sos1 expression (Figure S4B and Figures 3(h)–3(j)). The results indicate that sensitization can increase both RAS activation and Pak1 expression in mast cells. Inhibition of RAS blocks the Pak1 expression in mast cells. From the results, we may envisage a scenario in that sensitization increases RAS activation; the latter increases Pak1 expression; the Pak1 in turn interacts with Sos1 to promote RAS activation. Additionally, sensitization also increased Bcl2L12 expression in BMMCs that was abolished by knocking down the Pak1 expression. Overexpression of Pak1 in BMMCs also increased the Bcl2L12 expression in BMMCs (Figure S4C-D).

### 3.4. Semaphorin 3A (sema3A) Modulates the Pak1 Expression and RAS Activation in Mast Cells

**Figure 4:**
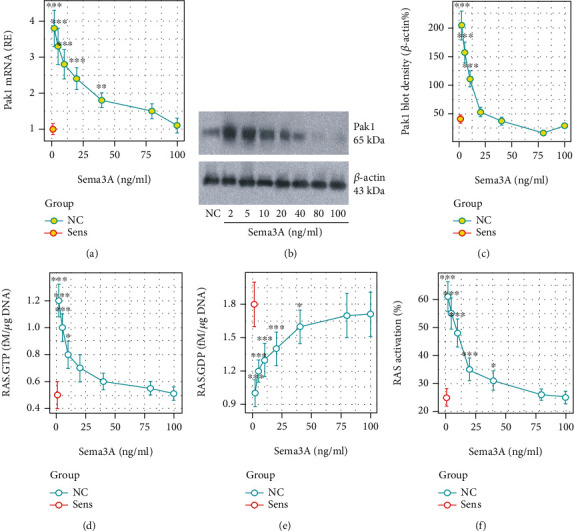
sema3A modulates Pak1 and RAS activities in sensitized mast cells. BMMCs were sensitized by incubating with anti-OVA-IgE (5 *μ*g/ml) overnight with or without the presence of sema3A at indicated concentrations. Cells were analyzed via RT-qPCR and RAS-specific ELISA. (a) mRNA levels of Pak1 in BMMCs. (b, c) Immunoblots show Pak1 protein levels in BMMCs. Curves show integrated density of immunoblots of Pak1. (d–f) Curves show levels of RAS.GTP (d), RAS.GDP (e), and RAS activation (f) in BMMCs. The data are presented as mean ± SD. ^∗^*p* < 0.05, ^∗∗^*p* < 0.01, ^∗∗∗^*p* < 0.001 (ANOVA+Dunnett's test), compared with the NC (naïve control) group. The data represent 3 independent experiments.

Published data indicate that sema3A can downregulate RAS activation by facilitating RAS.GTP to transform to RAS.GDP status and blocking the Ras/mitogen-activated protein kinase (MAPK) signaling pathway [18]. We then tested the effects of sema3A on modulating RAS activation and Pak1 expression in mast cells. Sensitized BMMCs were treated with sema3A at gradient concentrations in the culture overnight. The results showed that the presence of sema3A efficiently suppressed the expression of Pak1 (Figures 4(a)–4(c)), as well as the RAS activation (Figures 4(d)–4(f)) in BMMCs.

### 3.5. sema3A Restores Apoptosis Sensitivity of Sensitized Mast Cells

**Figure 5:**
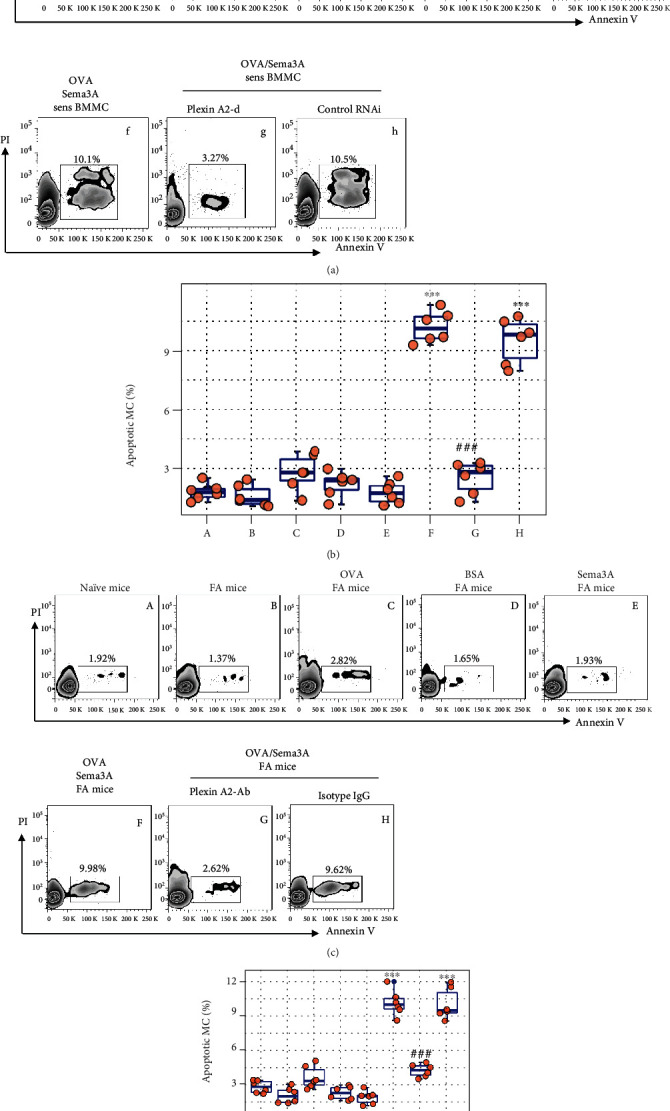
sema3A restores sensitivity to specific antigen-induced apoptosis of sensitized mast cells. (a, b) BMMCs were treated with the procedures denoted above each FCM panel. Gated FCM plots show apoptotic BMMC counts. Boxplots show summarized apoptotic BMMC counts of 6 independent experiments. Sens: sensitized; OVA: 5 *μ*g/ml; BSA: 5 *μ*g/ml; sema3A: 100 ng/ml; plexin A2-d: plexin A2-deficient BMMCs (generated by RNAi); control: BMMCs were treated with control RNAi reagents. (c, d) FA mice were treated with the procedures denoted above each FCM panel. Mast cells were gated first (presented in Figure S6), followed by counting apoptotic mast cells; gated plots show apoptotic mast cells; boxplots show summarized apoptotic mast cell counts of 6 mice per group. OVA: 100 *μ*g/mouse; BSA: 100 *μ*g/mouse; sema3A: 100 *μ*g/mouse; plexin A2-Ab: antiplexin A2-Ab (50 *μ*g/mouse/time, together with OVA challenge). The data of boxplots are presented as median (IQR). ^∗∗∗^*p* < 0.001 (ANOVA+Dunnett's test), compared with group A. ^###^*p* < 0.001 (Student *t* test), compared with group F.

We next tested a possible role of sema3A in restoring apoptosis sensitivity in sensitized mast cells. BMMCs were sensitized via exposure to anti-OVA IgE in the culture (Figure S3). Sensitized BMMCs were exposed to OVA (the specific antigen) with or without the presence of sema3A in the culture overnight. We found that exposure to OVA slightly induced sensitized BMMC apoptosis that was significantly enhanced by the presence of sema3A; knocking down the plexin A2 by RNAi (Figure S5), the receptor of sema3A, abrogated such an effect. Treating sensitized BMMCs with sema3A alone did not induce apoptosis (Figures 5(a) and 5(b)). We next fed OVA-sensitized FA mice with OVA or/and sema3A daily for 2 days. After the sacrifice, the small intestine was excised; LPMCs were isolated and analyzed by FCM. The results showed that administration of OVA alone induced about 2.82 ± 0.93% mast cell apoptosis, while administration of both OVA and sema3A induced about 10.3 ± 0.79% mast cell apoptosis that was abolished by concurrent administration of antiplexin A2- (the receptor of sema3A) Ab. Administration of BSA or sema3A alone did not apparently alter mast cell apoptosis in the FA intestine (Figure S6 and Figures 5(c) and 5(d)). The results demonstrate that administration of sema3A can restore the sensitivity to apoptosis inducers in sensitized mast cells.

### 3.6. Administration of sema3A Potentiates Specific Immunotherapy in Experimental FA

**Figure 6:**
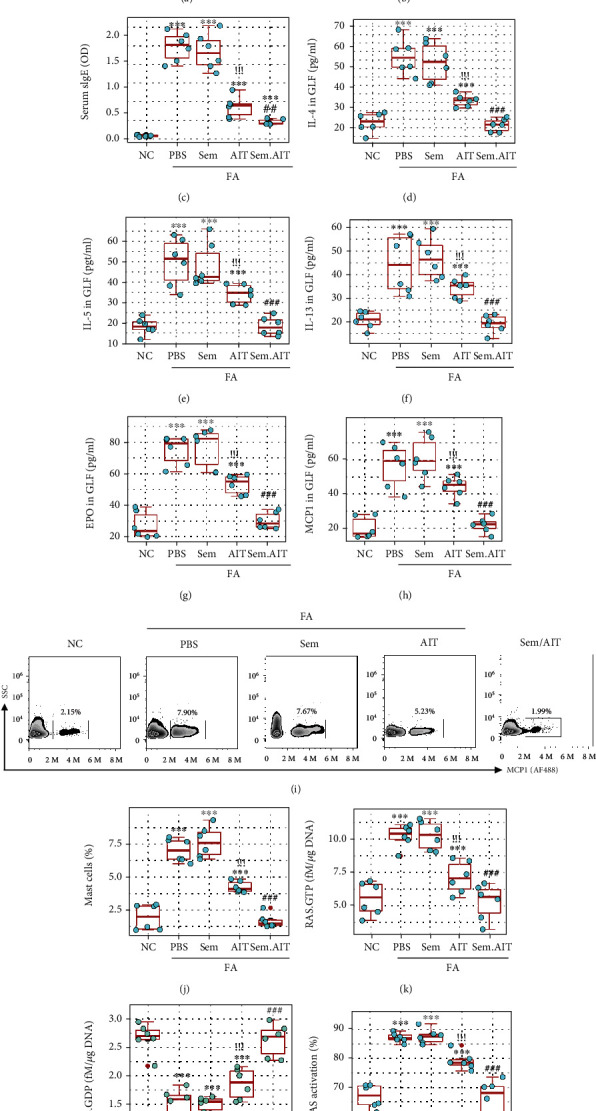
Assessment of sema3A effects on promoting AIT. FA mice were treated with sema3A (Sem, in short) or/and AIT or PBS. (a) Diarrhea mice during 2 h after allergen challenge. (b) Core temperature changes at 30 min after allergen challenge. (c) Serum allergen-specific IgE (sIgE) levels. (d–f) Gut lavage fluid (GLF) Th2 cytokine levels. (g, h) GLF EPO and mMCP1 levels. (i) Gated plots show mast cell frequency in LPMCs. (j) Summarized mast cell frequency in LPMCs. (k–m) RAS activation status in mast cells of the intestine. The data of boxplots are presented as median (IQR). ^∗∗∗^*p* < 0.001 (ANOVA+Dunnett's test) compared with the naïve control (NC) group. ^!!!^*p* < 0.001 (*t* test), compared with the PBS group. ^###^*p* < 0.001 (*t* test), compared with the AIT alone group. Each bubble presents data obtained from one mouse. The data of (i) are from one experiment that represent 6 independent experiments.

Finally, we developed an FA mouse model with ovalbumin (OVA) as the specific antigen (Figure S7). FA mice were treated with PBS (control), sema3A, or/and AIT. After completing the treatment, FA mice treated with PBS showed FA clinical symptoms (diarrhea and core temperature drop) (Figures 6(a) and 6(b)), increasing serum-specific IgE levels (Figure 6(c)), elevated Th2 cytokines (Figures 6(d)–6(f)), mast cell protease-1 (MCP1), eotaxin, and eosinophilic mediator EPO (eosinophil peroxidase) (Figures 6(g) and 6(h) and Figure S8) in gut lavage fluids (GLF). The frequency of mast cells and eosinophils in the intestinal tissues was increased (Figures 6(i) and 6(j) and Figure S8A-B). FA mice were treated with allergen-specific immunotherapy (AIT) with or without administration of sema3A. We found that administration of sema3A alone did not alter the FA response, but it significantly potentiated the AIT effects on the FA response (Figures 6(a)–6(f)). Mast cell frequency in the intestinal tissues and RAS activation of mast cells was also downregulated by the combination of sema3A and AIT (Figures 6(k)–6(m)). The results demonstrate that administration of sema3A can potentiate the AIT effects on alleviating experimental FA response.

## 4. Discussion

Our previous studies showed that FA mast cells had the apoptosis resistance feature [4]; the present study further revealed that higher RAS activation was detected in mast cells of FA mice and the sensitized BMMCs. RAS activation was positively correlated with the frequency of mast cells in the FA intestinal tissues and the apoptosis resistance in mast cells; this implicates a link between RAS activation and the mast cell aggregation in the intestine, in which the apoptosis resistance status played an important role. Further evidence showed that the higher Pak1 expression was positively correlated with the RAS activation levels in mast cells. The data showed that Pak1 promoted the RAS activation; the latter, in turn, promoted the Pak1 expression in mast cells. Inhibition of RAS by sema3A restored the sensitivity to apoptosis inducers in sensitized mast cells and promoted the therapeutic effects of AIT on experimental FA.

The mast cell aggregating in the lesion tissues is a pathological feature of allergic diseases, some chronic inflammations, and tumors. The underlying mechanism is not fully understood yet [19]. The present data also show a marked increase in mast cell frequency in the FA intestinal mucosa. Our data provide mechanistic evidence for this phenomenon. High RAS activation status was detected in FA mast cells. This phenomenon was reproduced by sensitizing BMMCs with sIgE in *in vitro* experiments. The RAS activation levels were positively correlated with the mast cell counts in the intestine, as well as with the apoptosis resistance in FA mast cells. Previous reports also mentioned that RAS was associated with the pathogenesis of allergic diseases; for example, Zhang et al. suggested that RAS activities were involved in several aspects of the pathophysiology of asthma, such as airway hyperresponsiveness, airway smooth muscle contraction, and airway remodeling [20]. Overexpression of phospholipase C*ε*, an effector of Ras and Rap small GTPases, can induce dermatitis [21]. The present data expand this knowledge slice by showing aberrantly high RAS activation in FA mast cells that is associated with the increase in mast cell number in the FA intestinal tissues.

Our previous studies [4] showed that mast cells isolated from intestinal tissues of mice with allergic inflammation showed higher expression of Bcl2L12; the present study expanded this by showing that mast cells isolated from allergic intestinal tissues expressed higher Pak1 levels. The Pak1 expression was positively correlated with the RAS activation levels in sensitized mast cells, as well as positively correlated with the mast cell frequency in the intestinal tissues. The data show that ablation of Pak1 abolished the sensitization-increased Bcl2L12 expression in mast cells. The fact suggests that signals of Pak1 are the upstream signals of Bcl2L12 contributing to the apoptosis resistance in sensitized mast cells. It is well known that the aberrant RAS activation plays a role in the sustaining overgrowth of cancer cells with a mechanism that RAS is stuck at the RAS.GTP status and does not transform to the RAS.GDP status [13]. Such a phenomenon was also detected in sensitized mast cells; the RAS.GTP levels were higher, and the RAS.GDP levels were lower in sensitized mast cells. This condition may contribute to the aberrantly higher expression of Pak1 in mast cells since inhibition of RAS abrogated the sensitization-induced Pak1 expression in mast cells.

Our previous studies showed that Bcl2L12 is bound to c-Myc, the transcription factor of FasL, to prevent the FasL expression. As FasL plays a critical role in the initiation of apoptosis [22], the presence of Bcl2L12 interferes with the apoptosis initiation. The present study revealed another aspect in the apoptosis resistance of mast cells. The higher expression of Pak1 is associated with this feature in mast cells of FA mice or the sensitized mast cells. Inhibition of Pak1 restores the sensitivity to apoptosis inducers in mast cells. As mast cell aggregation in the local tissues plays a critical role in allergy attacks, regulating the expression of Pak1 or using inhibitors to suppress Pak1 activities may be a novel remedy for use in the treatment of allergic diseases.

Son of Sevenless (Sos) is a key factor in RAS activation [23]. Our data show that Pak1 forms a triple complex with RAS and Sos1 in sensitized mast cells. The significance of this finding is that Pak1 involves the effects of Sos1 on RAS activation in mast cells. We also found that the sensitization-induced Pak1 expression in mast cells could be blocked by an RAS inhibitor, FTS, indicating that sensitization induces RAS activation prior to Pak1 expression. Taken together, the data suggest that RAS activation induces Pak1 expression, while Pak1 promotes RAS activation, and thus, it forms a sustaining circle; it confers mast cells' apoptosis-resistant feature.

The data show that inhibition of RAS restores the sensitivity to apoptosis inducers in sensitized mast cells. The apoptosis inducer of this study is the exposure to specific antigens, an event similar to the “activation-induced cell death” (AICD). The phenomenon of AICD was firstly found in T cells [24] that was also found lately in other cell types, such as cancer cells [25] and eosinophils [26]. We also observed it in a previous study [4] as well as in the present study. Thus, an activation is also an apoptosis inducer in mast cells. Because of the apoptosis resistance, sensitized mast cells do not respond well to apoptosis inducers, which may extend the lifespan of sensitized mast cells and, consequently, increases mast cell number in the local tissues; this is an interesting topic and needs to be further investigated.

It is well known that mast cells are one of the major effector cells in allergic diseases [1]. This statement is supported by the factors that administration of antihistamine agents or mast cell stabilizers can well control allergic symptoms [1]. Thus, antagonizing mast cell-released mediators or preventing mast cells from releasing mediators is the effective remedy to alleviate allergic symptoms. The present data show that RAS inhibition by sema3A restores the sensitivity to apoptosis inducers in mast cells. Administration of sema3A efficiently reduced mast cells in the intestine of FA mice. In other words, sema3A reduced the sources of allergic mediators in the FA mouse intestine and, thus, alleviated FA response. Others also noted that sema3A alleviated allergic diseases, such as allergic conjunctivitis and allergic rhinitis [12, 27]. The underlying mechanism may be that sema3A breaks the Pak1/RAS activation circle; restores the sensitivity of mast cells to apoptosis inducers, which reduces mast cells in the intestine; and, thus, contributes to alleviating FA symptoms.

The data showed that administration of sema3A not only reduced the mast cell number in the intestine but also attenuated the Th2 cytokines in the intestine and downregulated serum sIgE. This may result from the fact that the aberrant RAS activation is also involved in the development and maintenance of Th2 polarization in the intestine of FA mice. Others also noted this phenomenon; for example, Wu et al. found that fungal allergen could activate the RAS/RAF/ERK signal pathway to induce allergic inflammation in the airway tissues [28]. Mast cells express RasGRP4 (RAS guanine nucleotide-releasing protein-4); depletion of RasGRP4 markedly reduced experimental colitis (induced by feeding with dextran sodium sulfate) and arthritis [29]. Treating mice with allergic conjunctivitis with sema3A efficiently suppressed Th2 cytokine release in mice [12]. Another explanation of this phenomenon is that mast cell-derived IL-4 plays a critical role in the initiating Th2 polarization that was observed in previous studies [3]. The data show that after concurrent administration of sema3A and AIT, the mast cell number in the intestinal tissues was reduced, the mast cell mediators in GLF were decreased, and IL-4 in GLF was reduced as well. Whether these events are responsible for the reduction of Th2 response in FA mice needs to be further investigated.

We also observed that, after the treatment with both sema3A and AIT, the eosinophil number was reduced in the intestinal tissues; eosinophilic mediators were reduced in GLF. Previous reports also found that administration of sema3A inhibited eosinophils from entering the experimental allergic conjunctiva in mice [12]. This may be explained as because the mast cell number and activities of mast cells are suppressed by sema3A, the sources of eotaxin [30], one of the major chemotactic factors of eosinophils are attenuated; this contributes to reducing eosinophils in the intestine. Previous studies also observed that mast cell-deficient mice show less eosinophil infiltration in the lung in experimental airway allergy mice [31]. Whether sema3A can also regulate eosinophil activities needs to be further investigated.

Our data show that sensitization confers mast cells the apoptosis resistance. Previous studies also suggest that aggregation of Fc*ε*RI on the surface of mast cells by specific antigen promotes mast cell proliferation and survival [32, 33]. One of the proposed mechanisms is that sensitization increases Bcl2 family in mast cells. The Bcl2 family belongs to inhibitors of apoptosis. Our previous studies and current data also show that mast cells from sensitized mast cells express Bcl2L12, a member of the Bcl2 family. As a result, the sensitized mast cells gain the apoptosis resistance. This may extend the lifespan of mast cells and, consequently, increases the number of mast cells in the local tissues.

In summary, the present data show that sensitized mast cells are at a higher activation status and express higher levels of Pak1. The RAS activation and Pak1 expression mutually potentiate each other, sustaining the development and maintenance of apoptosis resistance in sensitized mast cells. Administration of sema3A can markedly promote the effects of AIT on alleviating experimental FA, suggesting that sema3A has the translation potential in the treatment of FA and other allergic disorders.

## Data Availability

All the data are included in this paper and the online supplemental materials.

## Abbreviations

FA: Food allergy
Pak1: p21-activated kinase-1
sema3A: Semaphorin 3A
FCM: Flow cytometry
PI: Propidium iodide
OVA: Ovalbumin
GLF: Gut lavage fluids.

## Supplementary Materials

Supplementary Materials The supplemental figures and experimental procedures of mice, generation of bone marrow-derived mast cells, flow cytometry, real-time quantitative RT-PCR, Western blotting, enzyme-linked immunosorbent assay, knockdown of Bcl2L12 expression in BMMCs by RNA interference, and overexpression of Bcl2L12 in BMMCs are presented in the online supplemental materials.
