# Supplementary material for: Semaphorin-3 Promotes Specific Immunotherapy Effects on Experimental Food Allergy
Source: J Immunol Res. 2022 Jun 19;2022:5414993. doi: 10.1155/2022/5414993 (PMC9234049; doi:10.1155/2022/5414993)
Supplement: Supplementary Materials — The supplemental figures and experimental procedures of mice, generation of bone marrow-derived mast cells, flow cytometry, real-time quantitative RT-PCR, Western blotting, enzyme-linked immunosorbent assay, knockdown of Bcl2L12 expression in BMMCs by RNA interference, and overexpression of Bcl2L12 in BMMCs are presented in the online supplemental materials. [file 5414993.f1.docx]

**Supplemental materials**

**Reagents**

Plexin A2 shRNA kit, Sos1 shRNA kit, Farnesyl thiosalicylic acid (FTS), antibodies (Ab) of plexin A2 (A-2), Sos1 (A-9), FcεRI (H-5, AF546), CD117 (E-3, AF594), MCP1 (CC1, AF488) and MBP (also called EMBP; F-6, AF594) were purchased from Santa Cruz Biotech (Santa Cruz, CA). Mouse recombinant Sema-3A fused to mouse Fc fragment was purchased from R&D Systems (Minneapolis, MN). Annexin v kit, propidium iodide (PI), compound 48/80 (C48/80) and ovalbumin were obtained from Sigma-Aldrich (St. Louis, MO). ELISA kits of IL-4, IL-5, IL-13, IFN-γ, mMCP1 and eotaxin were purchased from R&D Systems (Minneapolis, MN). Pak1 Ab, ELISA Kits of Ras GTPase and Ras GDP were purchased from abcam (Cambridge, MA). Bcl2L12 Ab, materials and reagents for immunoprecipitation, RT-qPCR and Western blotting were purchased from Invitrogen (Carlsbad, CA). The EPO ELISA kit was purchased from Dakewu Biomart (Beijing, China). OVA-specific mAb was purchased from Chondrex Inc. (Shanghai, China).

**Mice**

BALB/c mice were purchased from Beijing Experimental Animal Center (Beijing, China). Mice were maintained in a specific pathogen free facility at Zhengzhou University with accessing food and water freely. The animal experimental procedures were reviewed and approved by the Animal Ethical Committee at Zhengzhou University.

**Mast cell culture**

Purified mast cells were cultured in RPMI1640 medium for further experiments. The medium was supplemented with 10% fetal calf serum, 100 U/ml penicillin, 0.1 mg/ml, and 2 mM glutamine. Cell viability was 97%-99% as assessed by the Trypan blue exclusion assay.

**Assessment of cell apoptosis**

Cells were stained with propidium iodide (PI) and an annexin v kit following the manufacturer’s instructions, and analyzed by FCM. The annexin v^+^ cells, or PI^+^ annexin v^+^ cells were regarded as apoptotic cells.

**Generation of bone marrow-derived mast cells (BMMC)**

The bone marrows (BM) were flushed out the BALB/c mouse femurs with saline. Single BM cells were filtered through a cell strainer (40 µm) and cultured in RPMI1640 medium in the presence of IL-3 (30 ng/ml) and stem cell factor (10 ng/ml). The culture medium, including the reagents, was changed in every 3 days. About 3-4-week culture, BMMCs were purified by FCM with FcεRI and CD117 as the cell markers of isolation. The purity of isolated BMMCs was ranged in 96%- 99% as assessed by flow cytometry.

**Sensitization of BMMC**

BMMCs (5 × 10^5^ cells/ml) were cultured in the presence of anti-OVA mAb (50 ng/ml) for 2 h. Cells were washed with culture medium, and re-cultured in fresh medium. OVA (50 ng/ml) was added to culture medium to activate BMMCs. Supernatant was sampled 20 min and 30 min after the addition of OVA. Levels of histamine (in the 20 min-sample) and hexosaminidase (in the 30 min-sample) in supernatant were determined with purchased reagent kits following the manufacturer’s instruction.

**Flow cytometry (FCM)**

For the surface staining, cells were stained with fluorescence labeled antibodies or isotype IgG (diluted to 1 µg/ml) for 30 min at 4 °C. After washing with FCM buffer 3 times, cells (10^6^ cells/sample) were analyzed with a flow cytometer (BD FCMCanto II). In the intracellular staining, cells were fixed with 1% paraformaldehyde (containing 0.05% Triton-X100) for 1 h, and washed with phosphate-buffered saline 3 times; followed by the procedures of surface staining. The data were analyzed with a software package Flowjo (TreeStar Inc., Ashland. OR). The data obtained from isotype IgG staining were used as gating references.

**Real-time quantitative RT-PCR (RT-qPCR)**

Total RNA was extracted from mast cells with the TRIzol reagents, converted to cDNA with a reverse transcription kit following the manufacturer’s instructions. The samples were amplified in a qPCR device (DFX96, Bio Rad) with the SYBR Green Master mix in the presence of primers of Bcl2L12 (ttccgagttctatgccctgg and ccagtttacgatgcagagcc), or Pak1 (cctgaagttgtgacacgcaa and tctgggttctgaagctctgg). The results were calculated with the 2^-∆∆Ct^ method against the housekeeping gene β-actin.

**Western blotting**

Total proteins were extracted from purified FA intestinal mast cells, fractioned by SDS-PAGE, and transferred onto a PVDF membrane. The membrane was blocked by incubating with 5% skim milk for 30 min, stained with primary Abs (diluted to 200 ng/ml) overnight at 4 °C, washed with TBST (Tris-buffered saline containing 0.05% Tween 20) 3 times, incubated with HRP-conjugated second Abs for 2 h at room temperature, and washed with TBST 3 times. Immunoblots on the membrane were developed by the enhanced chemiluminescence and photographed in an imaging device (UVP, Cambridge, UK).

**Enzyme- linked immunosorbent assay (ELISA)**

Cytokine levels in culture supernatant or gut lavage fluids (GLF) were determined by ELISA with commercial reagent kits following the manufacturer’s instructions.

**Knockdown of plexin A2 or Sos1 expression in BMMCs by RNA interference**

BMMCs were prepared as described above, and treated with a plexin A2 or Sos1 shRNA reagent kit following the manufacturer’s instructions. The RNAi effects were checked by Western blotting 48 h after the treatment.

**Over expression of Pak1 in BMMCs**

The Pak1 expressing plasmids were constructed by the Sangon Biotech (Shanghai, China) with the whole length of the Pak1 gene sequence (Accession: NM_011035.2). BMMCs were transfected with the plasmids following the manufacturer’s instructions. The Pak1 over expression effects were checked by Western blotting 48 h later.

**Immunoprecipitation**

Proteins were extracted from sensitized BMMCs, and incubated with protein G agarose beads for 2 h to clear pre-existing immune complex. Samples were incubated with an anti-Pak1 Ab overnight to form immune complexes. Then, samples were incubated with protein G agarose beads to adsorb the immune complexes. The beads were collected by centrifugation (5,000 *g*, 10 min). Proteins on the beads were eluted with an eluting buffer, and analyzed by Western blotting. The membranes were stained with Abs of Pak1, Sos1, and KRAS, respectively.


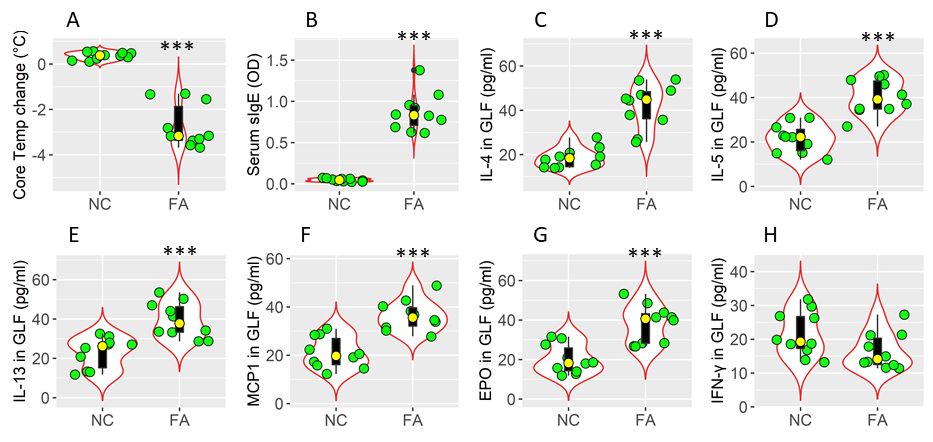


**Figure S1. Assessment of FA response in mice**. An FA mouse model was developed with OVA as a specific antigen. A, core temperature (Temp) changes at 30 min after OVA challenge. B, blood samples were collected at the sacrifice. The serum was isolated from blood and analyzed by ELISA. Violin plots show serum OVA-specific IgE (sIgE) levels. C-H, upon the sacrifice, a jejunal segment (15 cm) was excised, and rinsed with 1 ml PBS. The rinse solution was collected and designated gut lavage fluids (GLF). GLF was analyzed by ELISA. Violin plots show Th2 cytokines (C-E), representative allergic mediators (mMCP1 and EPO, F-G), and representative Th1 cytokines (IFN-γ, H). NC: Naïve control mice. Each group consists of 10 mice. The data of violin plots are presented as median (IQR). Each bubble in the plots presents data obtained from one mouse. ***, p<0.001 (Student *t* test), compared with the NC group.


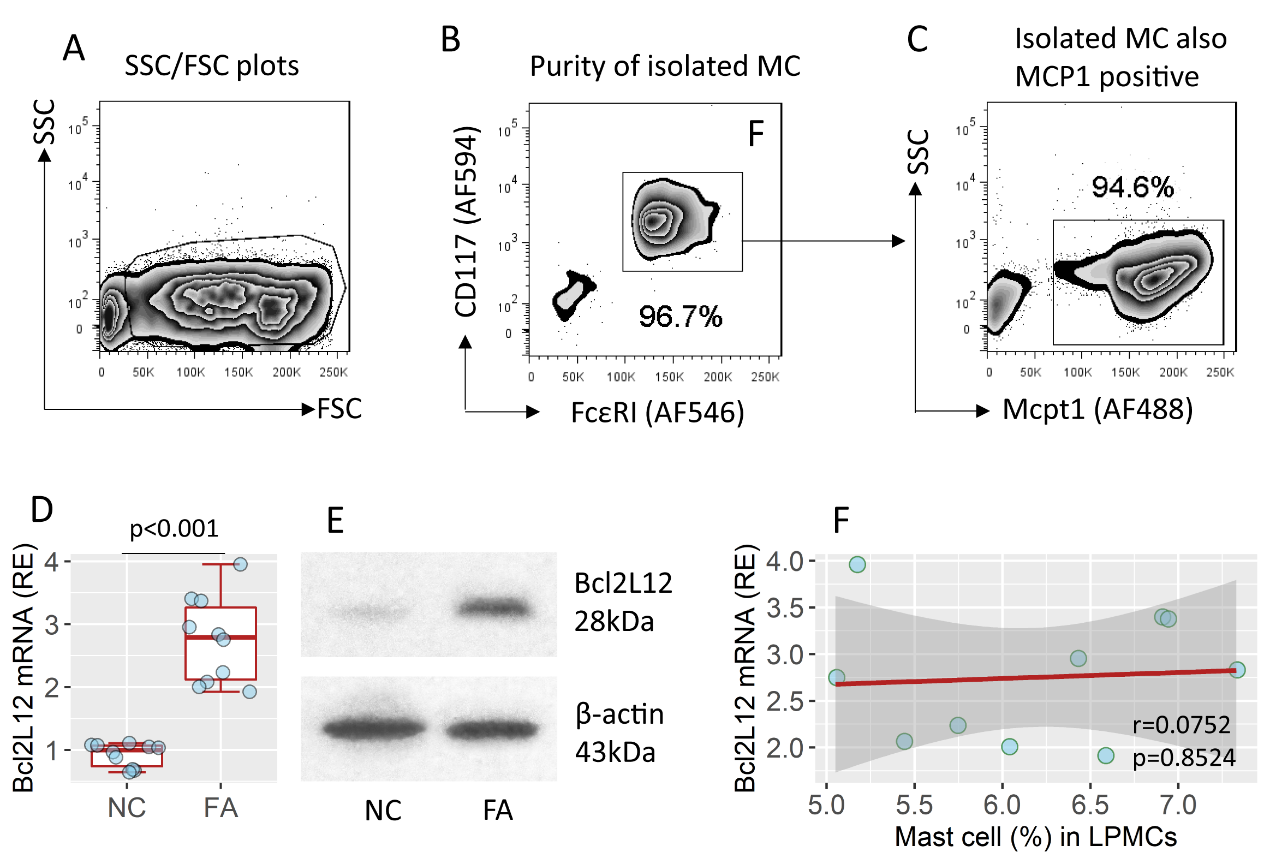


**Figure S2. Post-check of isolated mast cells**. Small intestinal segments were excised from mice upon the sacrifice. Lamina propria mononuclear cells (LPMCs) were isolated from intestinal tissues. A-C, mast cells were isolated from LPMCs by FCM with CD117 and FcεRI as the mast cell markers. Purified mast cells were post-checked by FCM. A, the SSC/FSC plots. B, gated FCM plots show purity of isolated mast cells. C, gated cells indicate that isolated mast cells are Mcpt1 positive. D-E, RNA and protein were extracted from isolated mast cells, and analyzed by RT-qPCR and Western blotting. Boxplots show Bcl2L12 mRNA levels and immunoblots show Bcl2L12 protein levels in mast cells. F, correlation analysis results between mast cell frequency in LPMCs and Bcl2L12 mRNA in mast cells.


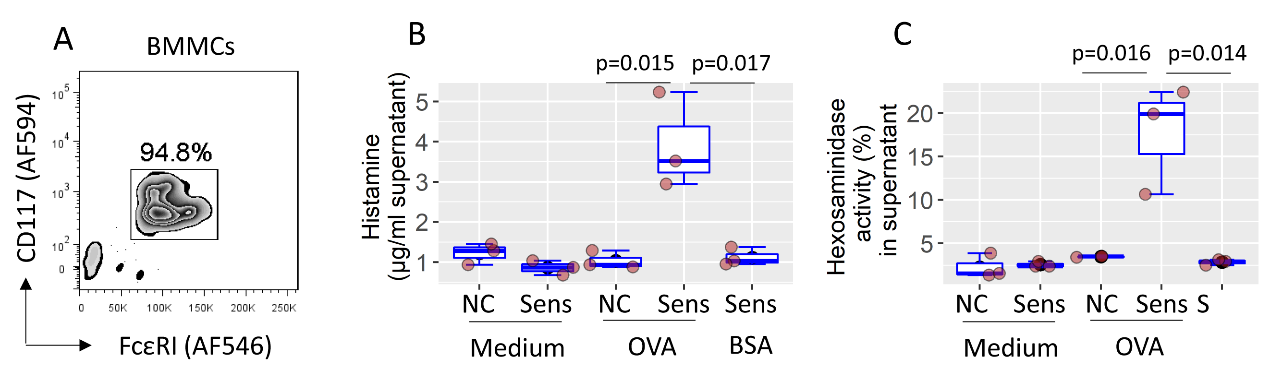


**Figure S3. Post-check of BMMCs**. BMMCs were prepared as described in the text, and purified by FCM with FcεRI and CD117 as the cell markers. A, gated cells are FcεRI^+^ CD117^+^ BMMCs. B-C, sensitized BMMCs were challenged with OVA (50 ng/ml) or BSA (50 ng/ml; an irrelevant antigen, used as a control) in culture as described above. Boxplots show the levels of histamine (B) and hexosaminidase (C) in culture supernatant.


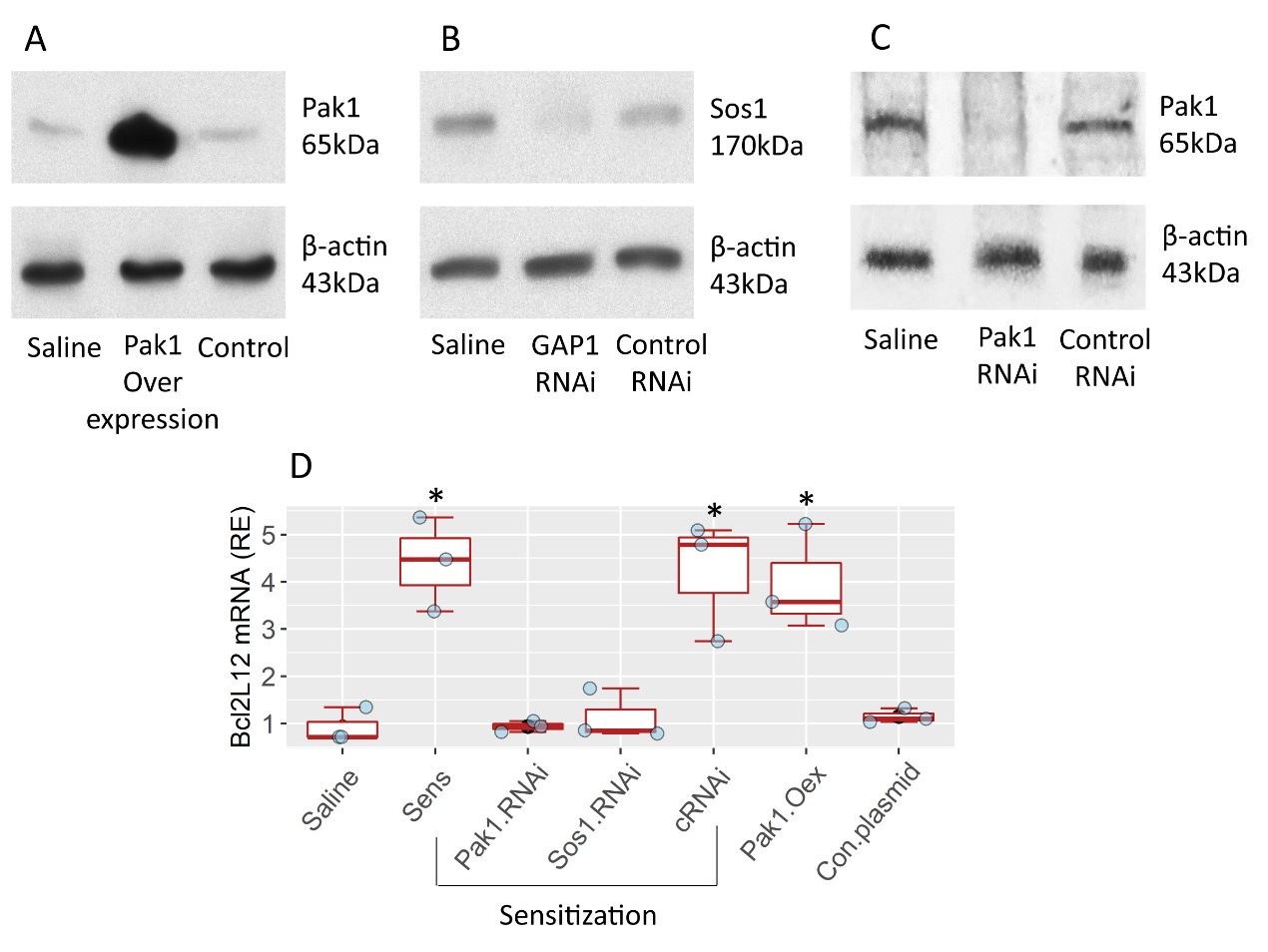


**Figure S4. Modulation of Pak1 and Sos1 expression in BMMCs alters Bcl2L12 expression**. BMMCs were treated with Pak1-expressing plasmids (A), or Sos1 RNAi reagents (B), or Pak1 RNAi reagents (C), following the manufacturer’s instructions. A-C, 48 h later, the expression of Pak1 or Sos1 was checked in the cells by Western blotting. The immunoblots show the protein levels of Pak1 or Sos1 in BMMCs. D, RNAs were extracted from BMMCs after the treatment of panels A-C, and analyzed by RT-qPCR. Boxplots show the Bcl2L12 mRNA levels. The data represent 3 independent experiments.


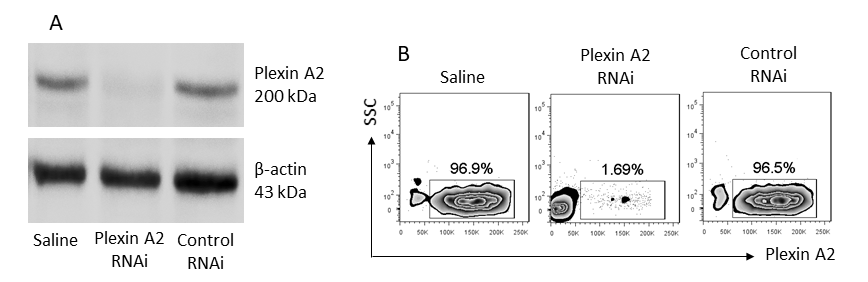


**Figure S5. Knockdown of plexin A2 in BMMCs**. BMMCs were treated with plexin A2 shRNA kit following the manufacturer’s instruction. Forty-eight hours later, the cells were analyzed by Western blotting and FCM. A, immunoblots show Plexin A2 protein levels in BMMCs. B, gated FCM plots show plexin A2^+^ BMMC frequency. The data represent 3 independent experiments.


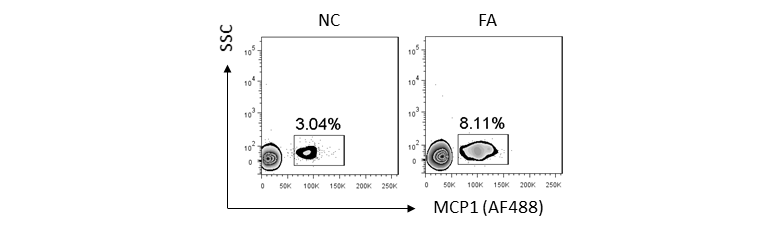


**Figure S6. Gating mast cells from LPMCs**. LPMCs were prepared from the intestine of NC mice and FA mice, and analyzed by FCM. Gated plots are mast cells, that were further analyzed to count apoptotic mast cells in Fig. 5C in the text.


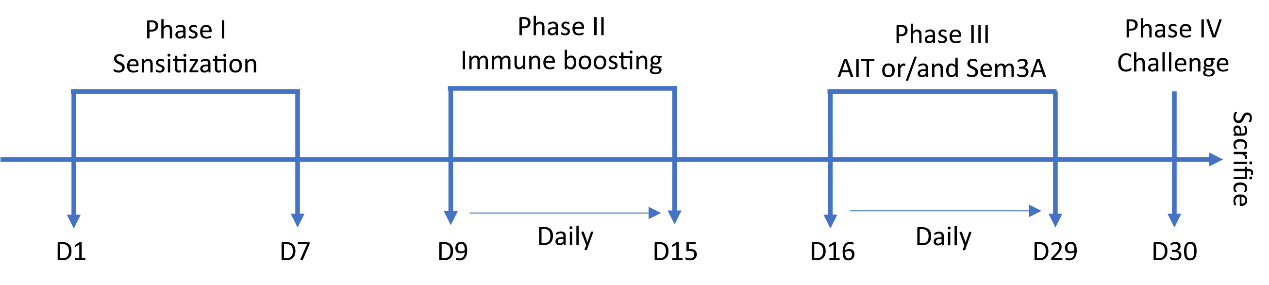


**Figure S7. Development of an FA mouse model and assessment of FA response**. BALB/c mice were used for this study. Phase I: Mice were subcutaneously injected with ovalbumin (OVA, 100 μg/mouse, mixed in 0.1 ml Alum) on the back skin on D1 and D7, respectively. Phase II: Mice were boosted by gavage-feeding with OVA (1 mg/mouse, in 0.3 ml saline) daily from D9 to D15. Phase III: Mice were treated with AIT or/and Sem3A (100 µg/mouse) daily from D16 to D29. Control FA mice were treated with PBS. On D30, mice were challenged by gavage- feeding with 0.3 ml OVA solution (50 mg /ml). Core temperature was measured in each with a rectal thermometer 30 min after oral challenge. Diarrhea was recorded during a 2-h-period after the challenge. Upon the sacrifice, a jejunal segment of 15 cm was excised, and rinsed with a syringe containing 2 ml PBS; the rinse fluids were collected at the other end of the jejunal segments, and used as the gut lavage fluids (GLF). Cytokine levels in GLF were determined by ELISA.


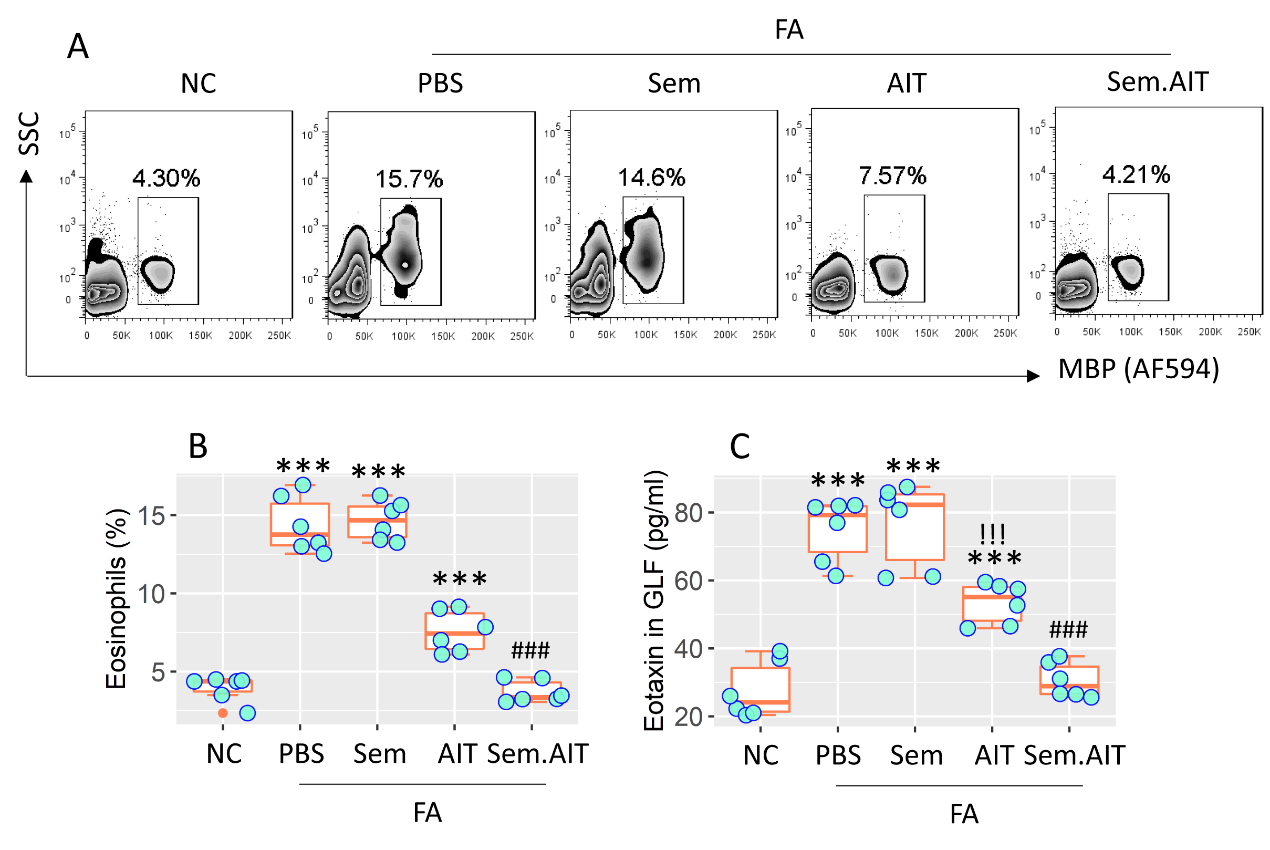


**Figure S8. Eosinophil frequency in LPMCs and GLF eotaxin levels**. The experimental procedures are the same as that of Fig. 6. A, gated FCM plots show eosinophil frequency in LPMCs. B, summarized eosinophil frequency in LPMCs of 6 mice per group. C, GLF eotaxin levels. The data of boxplots are presented as median (IQR). ***, p<0.001 (ANOVA + Dunnett’s test) compared with the naïve control (NC) group. !!!, p<0.001 (*t* test), compared with the PBS/FA group. ###, p<0.001 (*t* test), compared with the AIT alone group. Each bubble presents data obtained from one mouse. The data of panel A are from one experiment, that represent 6 independent experiments. Sem: Sema3A.
